# Subgroups of patients with late onset schizophrenia-like psychoses revealed by the analysis of glutathione-dependent enzymes and inflammation markers

**DOI:** 10.1192/j.eurpsy.2024.707

**Published:** 2024-08-27

**Authors:** T. Prokhorova, I. Boksha, L. Androsova, E. Tereshkina, V. Pochueva, O. Savushkina

**Affiliations:** ^1^FSBSI “MENTAL HEALTH RESEARCH CENTRE”, Moscow, Russian Federation

## Abstract

**Introduction:**

While chronic inflammation and enhanced imbalance of pro- and antioxidant, including glutathione-dependent, systems contribute substantially to pathogenesis of mental disorders in old age, extent of oxidative stress and degree of inflammatory processes severity are varying among patients with late onset schizophrenia.

**Objectives:**

Revealing various phenotypes in patients with late onset schizophrenia basing on measurement of activity levels for blood glutathione-dependent enzymes and inflammation markers and analysis of their links with clinical features of the patients.

**Methods:**

Of 59 studied women patients 34 were with late onset (after 40 years) and 25 with very late onset (after 60 years) schizophrenia or schizophrenia-like psychoses (F20; F22.8; F25; F23; F06.2 by ICD-10). 34 mentally healthy women elder than 50 years comprised controls. Glutathione reductase (GR), glutathione-S-transferase (GST), neutrophil elastase (NE), and α1-roteinase inhibitor (α1-PI) activities were measured in blood. PANSS, CDSS and CGI-S were used to assess the severity of psychotic symptoms, depression and treatment effectiveness.

**Results:**

In the whole group of patients, GR was lower (*p*<0.05), and α1-PI was higher (*p*<0.0001) than in control group. Clustering the patients by their biochemical and immunological signs revealed two clusters (C1, n=34, and C2, n=25) significantly differing by GST (*p*<0.0001), NE (*p*<0.0001), and α1-PI (*p*<0.001) activities. As compared with controls, GST and α1-PI were higher (*p*<0.05 and *p*<0.0001), and NE was lower (*p*<0.05) in C1. As compared with controls, GR activity was lower (*p*<0.05), NE activity was higher (*p*<0.001), and α1-PI activity was much higher (*p*<0.001) in C2. Patients of C1 and C2 did not differ in age, diagnosis, severity of the disease, but differed in clinical features of the course of the disease: significantly more patients with very late onset schizophrenia (76%) were met in C1 (χ2=13.41, *p*<0.001). Also, different clinical-biological correlations were found in these clusters. Particularly, negative correlations of baseline NE activity with PANSS general psychopathology subscale scores (R=-0.39, *p*<0.05) and with total PANSS scores (R=-0.39, *p*<0.05) were found in C1. Positive correlation of GST activity with PANSS positive subscale score was found in C2 (R=0.43, *p*<0.05).

**Conclusions:**

The revealed clusters differ in the extent of the glutathione antioxidant system impairment and in levels of the immune response markers. The revealing of the patient subgroups on the basis of biological markers reflecting impairments in metabolic and immune systems can represent interest in the search for individual treatment approaches.

**Disclosure of Interest:**

None Declared

